# Bromelain effectively suppresses Kras-mutant colorectal cancer by stimulating ferroptosis

**DOI:** 10.1080/19768354.2018.1512521

**Published:** 2018-08-30

**Authors:** Sujeong Park, Jinjoo Oh, Minhee Kim, Eun-Jung Jin

**Affiliations:** Department of Biological Sciences, College of Natural Sciences, Wonkwang University, Iksan, Chunbuk, Korea

**Keywords:** Colon cancer, bromelain, miRNA, ACSL-4, ferroptosis

## Abstract

Here, we investigated the possible anti-cancer properties of bromelain in Kras mutant human colorectal carcinoma cell lines and a mouse model harboring a Kras mutation. Cell growth and proliferation were significantly reduced in the Kras mutant colorectal carcinoma cell lines following treatment with 50 μg/mL bromelain as assessed by crystal violet staining and a proliferation assay. To identify the molecules responsible for this action, the expression levels of genes involved in signaling pathways and miRNAs were analyzed by real-time PCR. Among the genes tested, down-regulation of ACSL-4 and up-regulation of miRNAs targeting ASCL-4 were observed in Caco_2_ cells. Compared to the Kras wild-type colorectal carcinoma cell lines, Kras mutant colorectal carcinoma cell lines exhibited a remarkably up-regulated expression of ACSL-4, which is responsible for ferroptosis sensitivity. Moreover, the knockdown of ACSL-4 by a specific shRNA inhibited erastin-induced ferroptosis in Kras mutant DLD-1 cells as assessed by propidium iodide staining and lipid reactive oxygen species measurement. Our findings indicate that bromelain effectively exerts cytotoxic effects in Kras mutant colorectal cancer cells compared to in Kras wild-type colorectal cancer cells. Differential expression of ACSL-4 is responsible for the differential action of bromelain in regulating ferroptotic cell death.

## 1. Introduction

Colorectal cancer (CRC) is an important health problem worldwide. In South Korea, approximately 24,000 people are newly diagnosed with CRC each year (Kim 2013); approximately half of these patients die of the disease, making CRC the fourth leading cause of cancer death in South Korea. In most cases, CRC develops because of the progressive accumulation of genetic and epigenetic alterations that cause transformation and progression of normal colorectal mucosa to adenoma and, eventually, carcinoma (Coppede 2014). Particularly, mutations in the Kirsten Rat Sarcoma (K-Ras) gene are found in 30–50% in colorectal cancers (Castagnola and Giaretti 2005; Liu et al. 2011), which play an important role in the multi-step process of carcinogenesis. Moreover, compared to all other patients, patients with Kras mutation show a poorer prognosis and the G12D or G13D mutations have a significant adverse effect on outcomes compared to patients with other Kras mutations (Inoue et al. 2012).

Considerable evidence indicates that CRC is among the cancers that are most modifiable by diet (Birt and Phillips 2014). Dietary factors with convincing evidence for increasing CRC risk include red meat, processed meat, and alcoholic beverage and protective dietary factors include foods containing dietary fiber, garlic, milk, and calcium (Levi et al. 1999). Recently, attention has been focused on the possible role of plant food-derived natural products in preventing CRC (Rajamanickam and Agarwal 2008). Bromelain is a mixture of proteolytic enzymes (or proteases) derived from pineapple stem (*Ananas comosus* L., family Bromeliaceae) and numerous clinical studies have suggested the use of bromelain extracts in different areas, such as the food and pharmaceutical industries. Bromelain has been used to treat rheumatoid arthritis, oral inflammation, diabetic ulcers, rectal and perirectal inflammation, bronchitis, and sinusitis, among other conditions (Kane & Goldberg 2000; Brien et al. 2004; Pavan et al. 2012). Particularly, bromelain is known to enhance T-cell dependent immunity and attenuate allergic response (Secor et al. 2005). Bromelain attenuated inflammation in an asthma mouse model by reducing CD8^+^ T cell and CD19^+^ B cell numbers and lowering IL-13 concentrations (Secor et al. 2008). Recently, several studies suggested that bromelain has an anti-cancer effect by regulating multiple cellular and molecular targets (Chovotova et al. 2010). Bromelain significantly suppressed tumor initiation upon carcinogen exposure, as confirmed by a delay in the onset of tumorigenesis, as well as increased tumor-free survival in a mouse skin tumorigenesis model (Bhatnagar et al. 2015). Additionally, bromelain was found to increase cell death via apoptosis by the activation of caspase-cleaved cytokeratin in breast cancer cells (Dhandayuthapani et al. 2012) and via G2M arrest in human epidermoid carcinoma and melanoma cells (Bhui et al. 2012). Moreover, many *in vitro* studies showed that bromelain stimulates cell death of human cancer cell lines via alteration of Akt, ERK1, and ERK2 phosphorylation and mitogen-activated protein kinase (MAPK) pointing the involvement of these signaling pathway in the action of bromelain (Bhui et al. 2010; Romano et al. 2014)

However, the molecular mechanisms underlying the anti-cancer effects of bromelain are not fully understood. In this study, we evaluated the anti-cancer effects and underlying mechanism of bromelain in Kras wild-type and Kras mutant cells.

## 2. Materials and methods

### 2.1. Cell culture

Kras wild-type (Caco_2_; NCI-H508) or mutant (HCT116, G13D; DLD1, G12D) human colorectal cancer cell lines were grown as monolayers and treated with 50 μg/mL bromelain or 5 μM erastin for 12–24 h. Culture media used were; Caco_2_ in MEM (Gibco) supplemented with 20% FBS and HCT-116 and DLD1 and NCI-H508 in RPMI-1640 (Gibco) with 10% FBS with 1% antibiotic/anti-mycotic. Human normal colon fibroblasts CCD18co (CRL-1459, ATCC, Manassas, VA, USA) were grown in Eagle's Minimum Essential Medium (Gibco) supplemented with 10% fetal bovine serum, 1% antibiotic/anti-mycotic, 2 mM GlutaMAX, and 0.1 mM non-essential amino acids (Invitrogen).

### 2.2. Animals

KRAS^G12D^ mutant heterozygous mice (mouse strain was kindly gifted from Dr. Soo-Youl Kim at National Cancer Center in Korea) were given *ad libitum* access to food and water and maintained at 21–23°C with a 12 h:12 h light-dark cycle. The genotype of KRAS mutant alleles were analyzed by PCR using genomic tail DNA. The common forward primer sequence was 5′-TGCACAGCTTAGTGAGACCC-3′, wild-type reverse 5′-GACTGCT CTCTTTCACCTCC-3′, and mutant reverse 5′-GGAGCAAAGCTGCTAT TGGC-3′. Male mice were used for this study.

### 2.3. Carcinogenesis test

All experimental procedures were approved by the Animal Research Committee of Wonkwang University. Animals (*n* = 7) were given 2.5% DSS in drinking water for 5 days and then no treatment for 14 days as one cycle; this process was repeated for three cycles. In the last cycle, 2% DSS water treated to each group and no treatment for 14 days. During the three DSS cycle, 3 mg/kg bromelain were injected daily intraperitoneally and colon and spleen tissues were harvested after three DSS cycle in 57 days to study polyp burden and to perform histological staining.

### 2.4. Crystal violet staining

Cells were seeded at 10^4^ cells/well in 6-well plates, treated with bromelain, and then fixed with methanol. Cells were stained using 0.5% (w/v) crystal violet and extracted using 1% Triton X-100 (AMRESCO, Cochran Road Solon, OH, USA) in phosphate-buffered saline. The absorbance was read at a wavelength of 500 nm on a spectrophotometer (TECAN, Männedorf, Switzerland).

### 2.5. Lipid ROS staining

Cells were incubated with HCS LipidTOX Red neutral lipid stain (Thermo Fisher Scientific, Waltham, MA, USA) for 2 h at 37°C and stained with DAPI. Stained cells were visualized and captured with an Evos Fl Auto Cell Imaging System (Thermo Fisher Scientific).

### 2.6. Cell proliferation assay

Cell proliferation was performed using Quick cell proliferation colorimetric assay kit (Biovision # K301-500; Milpitas, CA, USA) according to the manufacturer’s instructions. Briefly, cells were seeded in 96-well cell culture plates and WST-1/ECS reagent was added, incubated for 4 h at 37°C, and then the absorbance was measured at 0, 6, 12, 18 h time point with a microplate reader at A460.

### 2.7. Immunohistochemistry for mice colon samples

Colon tissue was fixed in 10% (v/v) neutral buffered formalin 24 h and processed paraffin embedding. Paraffin-embedded colon tissues cut into 5 μm for H&E and immunostaining. Polyp number and length of submucosa layers was measured after scanned H&E stained slide based on their morphology. For immunohistochemistry, antigen retrieval was done using sodium citrate buffer (pH 6.0). anti-Ki67 antibody (Ab15580, Abcam) was incubated overnight, visualized with DAB substrate (Dako), and counterstained with hematoxylin. Histological images were acquired with a light microscope in the EVOS FL Auto imaging system (Thermo Fisher).

### 2.8. qRT-PCR

Total RNA was isolated from liver tissue using RNAiso Plus (TaKaRa) according to the manufacturer’s instructions. One μg RNA was reverse transcribed using the 5X All-In-One RT master mix (Abm). ACSL4 mRNA levels were amplified using specific primers; Forward: 5’-GCAGAGTACCCTGAAGGATTTG-3’, Reverse: 5’- CGTTGGTCTACTTG GAGGAATG-3’ with the Step One Plus real-time PCR systems (Applied biosystem). The relative expression level of a gene was normalized to the expression level of 18S rRNA.

### 2.9. Lentiviral siRNA vector and transfection

Lentiviral siRNA vector containing human ACSL4 (NM_004458, si hACSL4 490: GTTGAACTTCTGGAAAGTAAACTTAAGAC) were purchased from the Applied Biological Materials Inc., (ABM Inc.). Recombinant lentivirus was produced by transfecting HEK 293T cells using third generation packaging system and produced lentivirus was concentrated using Lenti-X concentrator (Takara). DLD-1 cell line was transfected with concentrated lentivirus in the presence of 4 μg/ml polybrene. After 24 h post infection, bromelain and erastin were treated.

### 2.10. Signaling pathway profiling

Signaling pathways were analyzed using signal transduction pathway finder RT^2^ profiler PCR Array (Qiagen, Hilden, Germany) according to the manufacturer’s protocol.

### 2.11. Mirna profiling

MicroRNA expression profiling was performed using the miScript System (miRNA PCR Array miFinder, Qiagen). The reverse transcription (RT) reaction was conducted using 1 μL miScript Reverse Transcriptase Mix, 4 μL 5x miScript RT Buffer, and 15 μL RNase-free water at 37°C for 60 min and the RT product was diluted into 100 μL. Quantitative real-time PCR was conducted using 10 μL SYBR Green PCR Master Mix, 2 μL miScript universal primer, 2 μL specific primer, 1 μL cDNA, and 5 μL RNase-free water for 45 cycles of 94°C for 15 s, 55°C for 30 s, and 70°C for 30 s using the ABI Step One Plus PCR system (Applied Biosystems, Foster City, CA, USA). MiRNA expression data were normalized to the expression levels of RNU6. Analysis and visualization of gene expression data were conducted using GenEx (Weihenstephan, Germany).

### 2.12. Statistical analysis

The data are mostly presented as the means ± SD. Significant differences between treatment and control values were analyzed by Student’s two-tailed *t*-test or one-way analysis of variance where appropriate. Differences were considered statistically significant if *P* < 0.05. Each variable was tested twice and experiments were repeated three times.

## 3. Results

### 3.1. Bromelain inhibits proliferation of Kras mutant CRC effectively via ACSL-4

**Figure 1.**
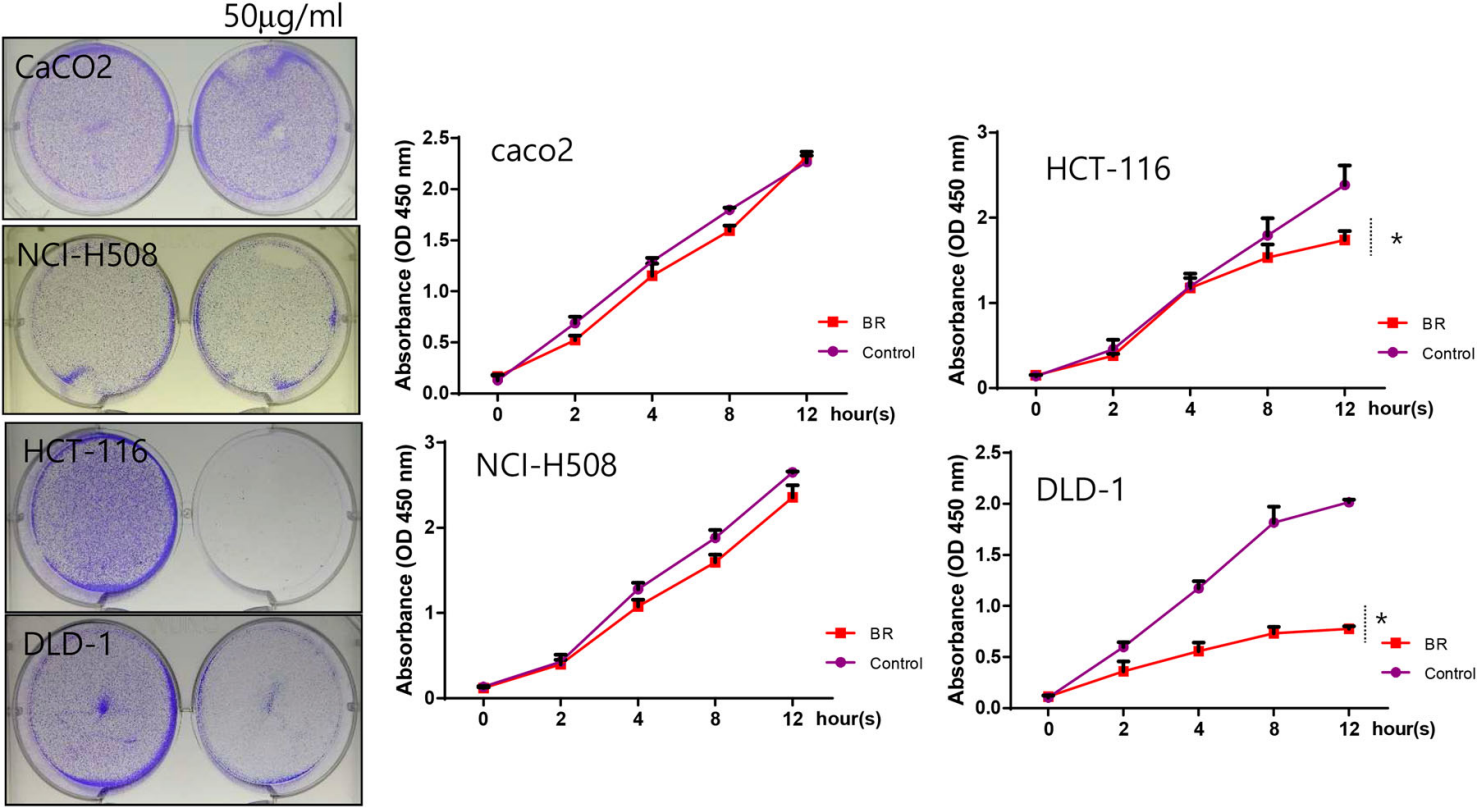
Bromelain effectively prevents proliferation of Kras mutant CRCs. Four different CRCs, CacO_2_, NCI-H508, HCT-116, and DLD-1, were cultured with 50 μg/mL of bromelain for 0, 2, 4, 8, and 12 h, stained with crystal violet (left panel), and assayed for cell proliferation (right panel). The results are shown as the mean, with error bars representing the 95% confidence interval (lower/upper limit); * *p* < 0.05.

**Figure 2:**
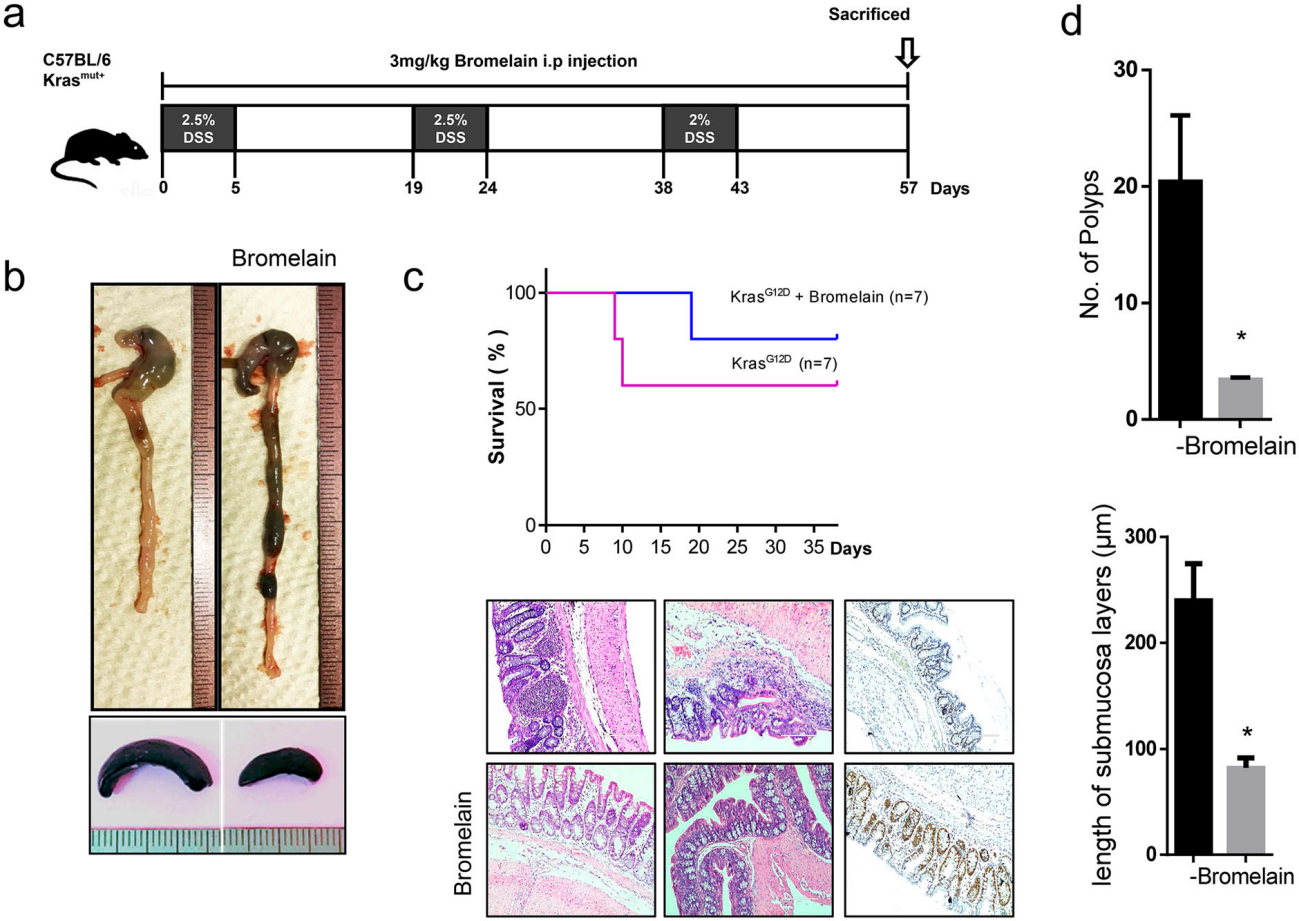
. Bromelain suppressed carcinogenesis in colon cancer mouse model. (a) Schematic diagram of experimental design. (b) Comparison of intestine and spleen in DSS-treated Kras mutant mice with or without bromelain treatment. (c) Comparison of survival rate (upper panel) and hematoxylin and eosin and Ki67 staining (lower panel) in DSS-treated Kras mutant mice with or without bromelain treatment. (d) Comparison of polyp number and submucosal layer length in DSS-treated Kras mutant mice with or without bromelain treatment.

To study the effects of bromelain on the proliferation of CRC cells, Kras wild-type (Caco_2_, NCI-H508) and Kras mutant (HCT-116, DLD-1) CRC cells were used. Cells were exposed to 50 μg/mL bromelain and their proliferation was analyzed at 0, 2, 4, 8 and 12 h. interestingly, severe suppression of cell proliferation was only observed in Kras mutant cells, i.e. HCT-116 and DLD-1 (Figure 1). Cell proliferation of Kras wild-type cells, i.e. CacO_2_ and NCI-H508, was not significantly affected by bromelain treatment. This suggests that bromelain effectively inhibits Kras mutant CRC cells. Furthermore, to confirm the inhibitory action of bromelain on colon cancer, we treated KRAS mutant mice (KRAS^mut/+^) with DSS in the absence or presence of bromelain (Figure 2). In the presence of bromelain, increased intestinal inflammation and enlarged spleen (Figure 2(b)) induced by DSS treatment were recovered. Moreover, the survival rate was significantly increased (Figure 2(c)) and the number of polyps and the length of submucosa layers were reduced (Figure 2(d)) by bromelain treatment in DSS-treated KRAS mutant mice.

**Figure 3.**
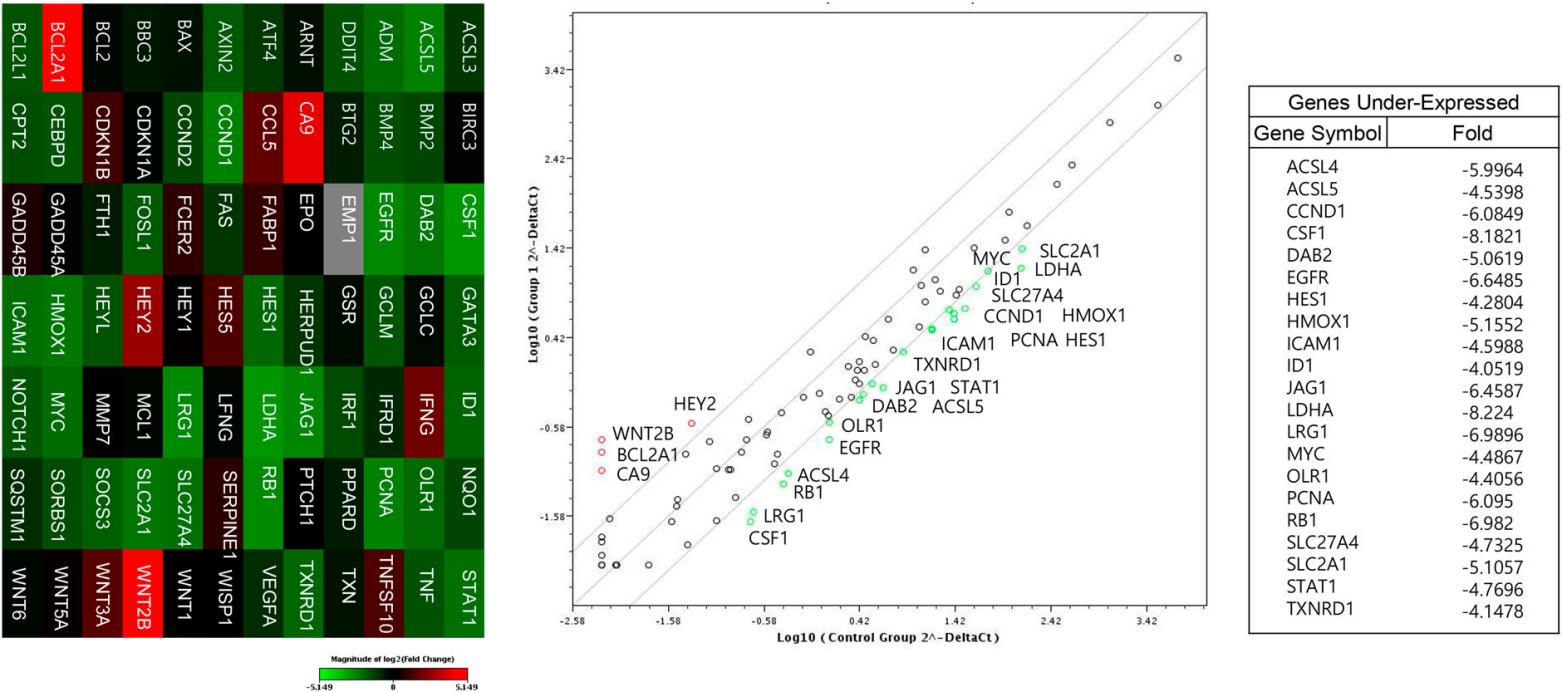
Differential induction of ACSL-4 by bromelain between Kras wild-type CRCs and Kras mutant CRCs. (a) CacO_2_ and DLD-1 CRCs were treated with 50 μg/mL of bromelain and analyzed for the expression levels of genes involved in cell signaling pathways.

**Figure 4.**
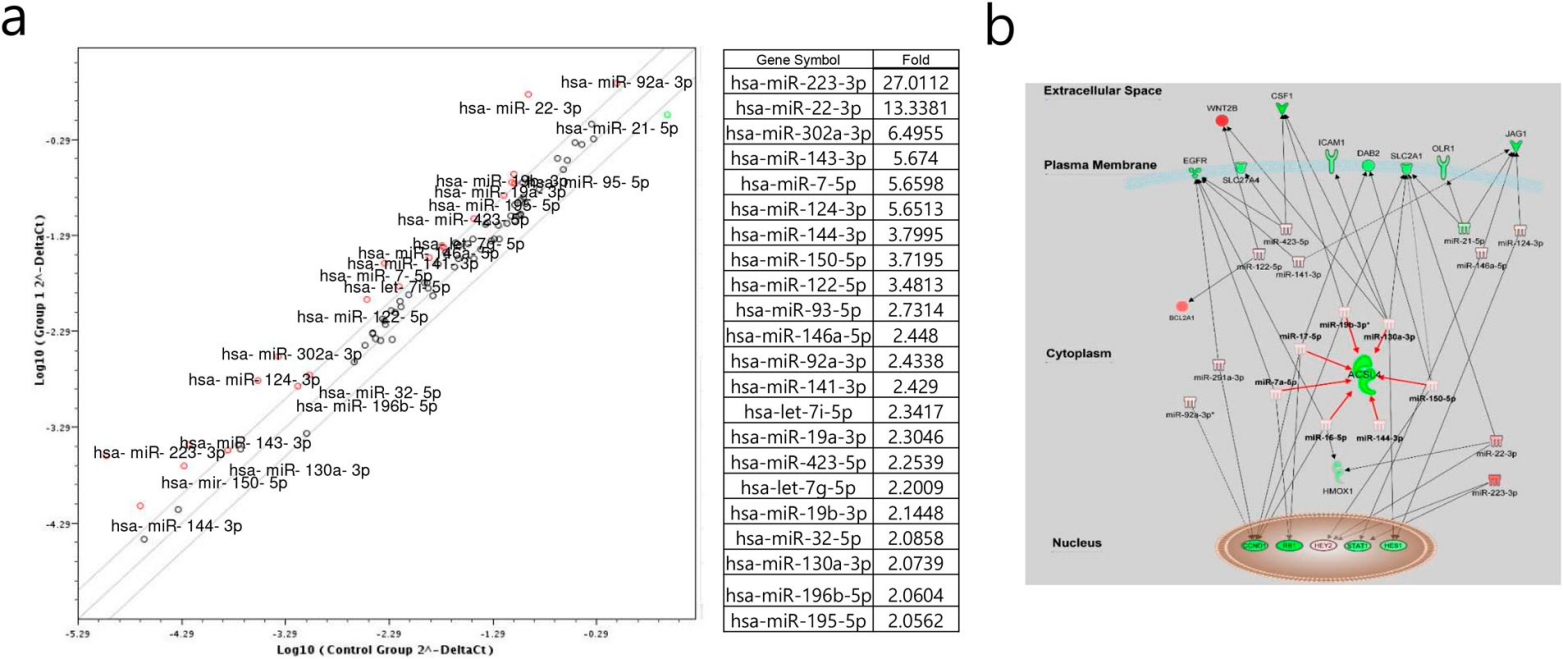
Differential induction of ACSL-4 by bromelain between Kras wild-type CRCs and Kras mutant CRCs. (a) CacO_2_ and DLD-1 CRCs were treated with 50 μg/mL of bromelain and analyzed for the expression levels of genes. The results are presented as fold-change of the expression levels of miRNAs in DLD-1 cells. (b) Interactome between genes and miRNAs whose expression levels were changed in CacO_2_ cells compared to in DLD-1 cells was presented by IPA.

To elucidate the underlying mechanism of these effects, we analyzed the expression levels of genes involved in cell signaling pathways (Figure 3) and miRNAs (Figure 4) using CaCO_2_ and DLD-1 cells treated with bromelain. The genes ACSL4, ACSL5, CCND1, CSF1, DAB2, EGFR, HES1, HMOX1, ICAM1, ID1, JAG1, LDHA, LRG1, MYC, OLR1, PCNA, RB1, SLC27A4, SLC2A1, STAT1, and TXNRD1 were significantly down-regulated in CaCO_2_ cells compared to in DLD-1 cells and the miRNAs miR-223-3p, -22-3p, -302a-3p, -143-3p, -7-5p, -124-3p, -144-3p, -150-5p, -122-5p, -93-3p, -146a-5p, -92a-3p, 141-3p, -423-5p, 19b-3p, 32-5p, 130a-5p, 196b-5p, and 195-5p were significantly up-regulated in CaCO_2_ cells compared to in DLD-1 cells. As shown in Figure 4(b), interactome analysis between these down-regulated gene and up-regulated miRNAs altered by bromelain in CaCO_2_ cells compared to in DLD-1 cells indicated that ACS-4 is a key regulatory molecule; elevated miR-19b-3p, -130a-3p, -150-5p, -144-3p, -16-5p, -7a-5p, and -17-5p in bromelain-treated CaCO_2_ cells compared to in DLD-1 cells potentially targeted ACSL-4 and resulted in suppression of ACSL-4.

### 3.2. Bromelain induces ROS-induced ferroptosis in Kras mutant CRC cells via ACSL-4

**Figure 5.**
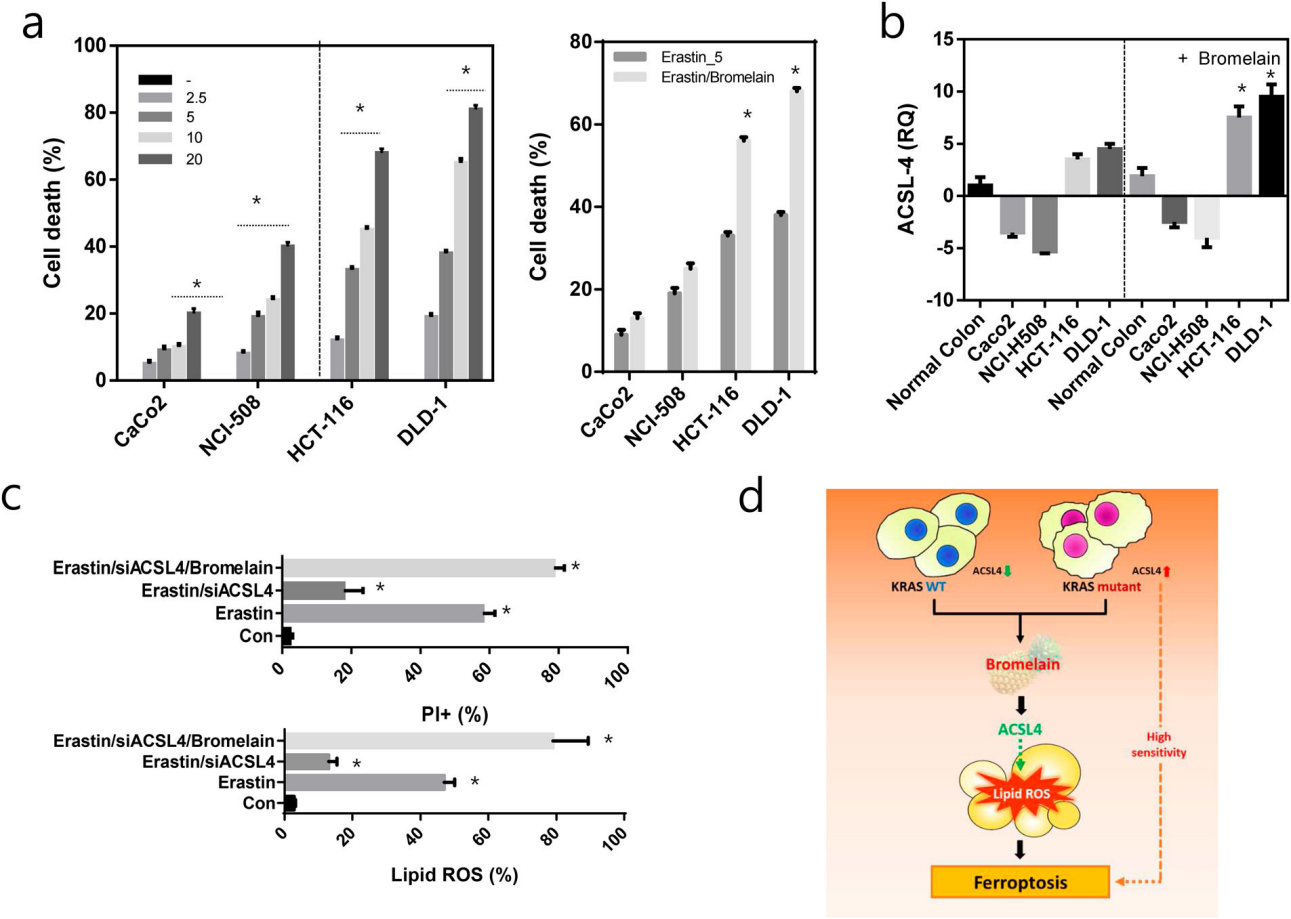
ACSL-4 induced ferroptosis. (a) Four different CRCs, CacO_2_, NCI-H508, HCT-116, and DLD-1, were cultured with different concentrations of erastin (left panel) or 5 μM erastin with or without bromelain (right panel) and cell death was analyzed. (b) Four different CRCs, CacO_2_, NCI-H508, HCT-116, and DLD-1, and normal colon cells, CCD18co, were treated with or without bromelain and the expression level of ACSL-4 was analyzed. (c) DLD-1 cells were treated with erastin, siACSL-4, or bromelain. Cell death was quantified by PI staining coupled with flow cytometry (upper panel) and lipid ROS was measured (lower panel). (d) Schematic diagram describing the action of bromelain in ferroptosis. The results are shown as the mean, with error bars representing the 95% confidence interval (lower/upper limit); * *p* < 0.05.

We first assayed cell viability in four CRC cells after treatment with the ferroptotic inducer erastin at 2.5–20 mM for 24 h. Compared to Kras wild-type (Caco_2_, NCI-H508) cells, Kras mutant CRC (HCT-116, DLD-1) cells were sensitive to erastin-induced cell death (Figure 5(a)). Interestingly, these ferroptosis-sensitive Kras wild-type cells expressed relatively low levels of ACSL4 mRNA, whereas KRAS mutant CRC cells expressed significantly increased levels of ACSL4 mRNA (Figure 5(b)). Moreover, bromelain treatment induced ACSL-4 and increased erastin-induced cell death in Kras mutant CRC cells. Treatment of DLD-1 with erastin induced a significant increase in cell death as measured by propidium iodide (PI) staining coupled with flow cytometry (Figure 5(c)). Co-introduction of ACSL-4-specific siRNA (siACSL-4) with erastin suppressed cell death. However, this decrease in cell death was significantly increased by co-treatment with bromelain. ROS accumulation by erastin treatment, one of the hallmarks of ferroptosis, was reduced by co-introduction of siACSL-4, whereas this reduced ROS, by co-introduction of siACSL-4, was significantly increased by co-treatment with bromelain (Figure 5(c)).

## 4. Discussion

Bromelain is a pineapple stem extract with a variety of beneficial effects in many different diseases including gastrointestinal disease and several inflammatory diseases (Pavan et al. 2012). Here, we showed that bromelain has greater anti-proliferative effects on Kras mutant CRC cells compared to Kras wild-type CRC cells. Bromelain is known to have anti-proliferative effects and pro-apoptotic effects in different tumor cell lines including gastric carcinoma cells, ovarian cancer cells, and breast cancer cells (Dhandayuthapani et al. 2012; Amini et al. 2014; Romano et al. 2014). In Caco-2 cells, bromelain was shown to reduce cell proliferation and induce apoptosis via activation of caspase 3/7 (Romano et al. 2014). In this study, we observed that the anti-proliferative effect of bromelain on Caco-2 cells was not significant. However, we observed a significant reduction in the proliferation of HCT-116 and DLD-1 cells, two RAS mutant (KRAS^G13D^, KRAS^G12D^, respectively) cell lines, by bromelain, suggesting that bromelain acts in a Kras-dependent manner.

To explore the molecules responsible for and underlying mechanisms of the effects of bromelain in Kras-dependent anti-proliferative and apoptotic action, we analyzed the expression levels of genes involved in cell signaling pathways and various miRNAs. Our results showed that differential induction of ACSL-4 by bromelain may explain the increased level of ACSL-4 by bromelain in DLD-1 cells compared to in CaCo-2 cells.

ACSL-4 is an isozyme in the long-chain fatty-acid-coenzyme A ligase family and is involved in the conversion of free long-chain fatty acids into fatty acyl-CoA esters, and thus plays a key role in lipid biosynthesis and fatty acid degradation. Dysregulation of lipid metabolism contributes to various types of biological events, including cell death. Recently, Doll and colleagues (2017) suggested that ACSL4 is critical role for the induction of ferroptosis, a programmed form of necrotic cell death, and can predict sensitivity to ferroptosis by modulating phospholipids, specifically phosphatidylethanolamine. Therefore, induction of ACSL-4 by bromelain in Kras mutant cell lines may explain the highly sensitive in ferroptosis. Ferroptosis is driven by the loss of activity of lipid repair enzymes, which results in the accumulation of lipid-based ROS (Yang and Stockwell 2016). Here, we demonstrated that bromelain effectively causes ferroptotic cell death in Kras mutant cell lines compared to in Kras wild-type cells by modulating ACSL-4 levels (Figure 5(d)). The activation of ferroptosis by bromelain in Kras mutant CRCs may be therapeutically important for poorly responsive Kras mutant tumor cells.
